# Impact of Change in Neonatal Resuscitation Program Guidelines for Infants Born Through Meconium-Stained Amniotic Fluid

**DOI:** 10.3390/children12081072

**Published:** 2025-08-15

**Authors:** Hamza Abbasi, James Shelton, Praveen Chandrasekharan, Munmun Rawat

**Affiliations:** 1 Neonatology Department, John R. Oishei Children’s Hospital, Buffalo, NY 14023, USA; pkchandr@buffalo.edu (P.C.); munmunra@buffalo.edu (M.R.); 2 Neonatology Department, State University of New York at Buffalo, Buffalo, NY 14214, USA; jshelton@buffalo.edu

**Keywords:** meconium aspiration syndrome, meconium-stained amniotic fluid, endotracheal suctioning, neonatal resuscitation program

## Abstract

**Background**: In 2016, the neonatal resuscitation program (NRP) changed its recommendation to perform endotracheal suctioning in non-vigorous neonates born through meconium-stained amniotic fluid (MSAF). The objective of this study is to compare outcomes in non-vigorous neonates born through MSAF before and after the change in the NRP’s recommendations. **Methods**: This is a retrospective cohort study in a single center assessing all neonates ≥34 weeks of gestation with MSAF in 2010–2015 (pre-implementation of new guidelines) and 2017–2022 (post-implementation of new guidelines). **Results**: Neonates receiving tracheal suctioning were more likely to be diagnosed with MAS (29.3% vs. 19.7%; *p* = 0.03) and PPHN (8.9% vs. 2.5%; *p* = 0.003) and more likely to receive surfactant (7.6% vs. 3.2%; *p* = 0.03). **Conclusions**: In our institution, non-vigorous neonates born via MSAF after the change in NRP guidelines were less likely to be diagnosed with MAS and PPHN and were less likely to receive surfactant. Our study supports current NRP guidelines.

## 1. Introduction

Meconium-stained amniotic fluid (MSAF) can occur in up to 25% of all pregnancies, which often causes meconium aspiration syndrome (MAS), a potentially devastating outcome that can lead to surfactant inactivation, persistent pulmonary hypertension of the newborn (PPHN) or hypoxic respiratory failure (HRF) requiring mechanical ventilation in up to 50% of affected patients, and death in 5–12% (up to 40%) of affected patients [1,2].

The delivery room management of MSAF has evolved over the last two decades [3]. Prior to 2016, the neonatal resuscitation program (NRP) recommended intubating neonates to suction meconium below the level of the glottis in non-vigorous neonates [4]. However, in its two most recent editions, the NRP changed that recommendation [5,6]. This change was based on a small randomized-controlled trial by Chettri et al. that assessed differences in outcomes in non-vigorous neonates born via MSAF that received endotracheal suctioning vs. neonates that did not receive endotracheal suctioning; the study had 61 patients in each group and concluded there were no differences in the MAS incidence and severity as well as mortality and neurodevelopmental outcomes at 9 months of age between the two groups [7].

Conversely, an ovine study from our large animal lab at the University at Buffalo, where meconium aspiration was induced in asphyxiated newborn lambs, compared 14 lambs that did not receive suctioning to 15 lambs that received endotracheal suctioning; the study showed improved ventilation and oxygenation parameters in lambs that received tracheal suctioning [8]. After the change in NRP guidelines in 2015, a retrospective review by Kalra et al. observed a higher incidence of adverse outcomes such as MAS, PPHN, and the need for mechanical ventilation in their institute since endotracheal suctioning was discontinued; however, the study only included 69 patients in both epochs [1].

A retrospective study by Sheikh et al. found no difference in the MAS incidence or death rates before or after the change in guidelines in a cohort of 95 patients that received suctioning and 91 patients that did not receive suctioning [9]. A meta-analysis published in 2020 by Phattraprayoon et al. that included four studies with a total of 581 non-vigorous patients born via MSAF concluded that there was no difference in outcomes before and after the change in guidelines [9,10].

There is an ongoing debate about how to manage neonates born through MSAF due to conflicting results.

## 2. Materials and Methods

This is a single-center retrospective cohort study in a level IV 64-bed regional perinatal center. A chart review was conducted on all neonates born at ≥34 weeks of gestation during the years 2010–2015 and 2017–2022. All neonates admitted to mother–baby unit (MBU) and NICU were included.

This study was approved by the Institutional Review Board of the State University of New York at Buffalo, John R. Oishei Children’s Hospital (IRB ID STUDY00006961). For patients admitted to the NICU, data was extracted from our NICU’s NeoData database (Isoprime Corporation, Naperville, IL, USA) using a query function. Charts were reviewed individually to confirm data. For patients admitted to the MBU, delivery and admission notes of non-vigorous patients born via MSAF were reviewed. We included MBU patients in our cohort to compare initial Apgar scores and initial need for respiratory support.

**Figure 1:**
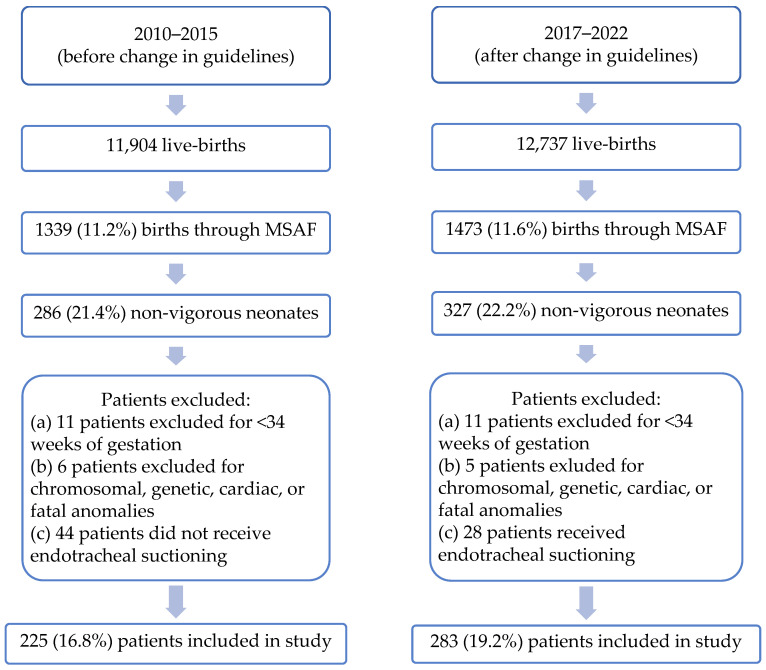
Inclusion/exclusion criteria.

Neonates born in 2016 were omitted from the analysis due to the transition period following the 2016 changes to the NRP guidelines. During this time, providers were still undergoing training, leading to inconsistencies and overlap in the use of suction versus no-suction practices of the endotracheal tube using a meconium aspirator (Neotech Meconium Aspirator^®^, https://www.neotechproducts.com/product/meconium-aspirator/, accessed on 4 June 2025) . We excluded neonates born ≤34 weeks of gestation; vigorous neonates; and neonates with chromosomal, genetic, cardiac, or fatal anomalies. We also excluded neonates that were not managed per existing NRP guidelines at the time of birth (non-vigorous neonates born via MSAF that did not receive tracheal suctioning prior to 2016 and non-vigorous neonates born via MSAF that did receive tracheal suctioning after 2016 change in guidelines) (Figure 1).

Data was electronically extracted from the NeoData NICU database, derived from documentation of events in the delivery room. NeoData is a searchable data system for Neonatology designed to assist in documentation of notes and daily patient management of our NICU patients and integrates with our hospital’s electronic medical record system [11]. It is used in approximately 100 NICUs in the USA. NeoData was queried for information on infants admitted to our NICU between 1 January 2010 and 31 December 2015 as well as 1 January 2017 and 31 December 2022. Query was limited to those infants with gestational ages ≥ 34 completed weeks gestation, who had meconium-stained fluid, and who were considered non-vigorous at birth. Delivery room notes in NeoData were queried for the following terms for ‘non-vigorous’: apneic, bradycardic, floppy, poor tone, hypotonic, gasping, poor respiratory effort, and no respiratory effort. Data and diagnoses from Table 1 and Table 2 were then extracted from the same query function.

Meconium aspiration syndrome was defined as respiratory distress with radiographic evidence of meconium aspiration (patchy infiltrates with hyperinflation) following a delivery through meconium-stained amniotic fluid.

Persistent pulmonary hypertension of the newborn was defined as hypoxic respiratory failure associated with the following echocardiographic findings: right ventricular hypertrophy, elevated right ventricular systolic pressure, right atrial dilatation, tricuspid/pulmonic valve regurgitation, right to left shunting at the level of patent ductus arteriosus/patent foramen ovale, and interventricular septal flattening or deviation.

Hypoxic–ischemic encephalopathy was defined as a disturbed neurologic function (decreased consciousness, seizures, apnea/respiratory insufficiency, depression of tone and neonatal reflexes) within the first 48–72 h in a ≥35-week infant exposed to a prenatal event causing hypoxia and ischemia.

In spite of the implementation of change in guidelines through 2016 to discontinue endotracheal suctioning, 28 patients in the second epoch received endotracheal suctioning; those were excluded from the study. Patients who did not receive endotracheal suctioning in the first epoch (44 patients) were excluded as well.

We hypothesized that the incidence of MAS and neonatal intensive care unit (NICU) admissions for respiratory distress would be similar between non-vigorous infants born through MSAF in the no-suction era and the prior routine suction era. We conducted an individual chart review of all infants born through MSAF during both periods/epochs.

Statistical Analysis: The categorical data was described using frequencies, and percentages were used to describe the categorical data. Groups were compared using the chi-square test. For normally distributed continuous variables, the means and standard deviation were used to describe the data. For continuous variables that were not normally distributed, medians with interquartile ranges were used to describe data. The groups were compared using the *t* test for continuous data. For all statistical tests, a *p* value less than 0.05 was considered statistically significant.

## 3. Results

A total of 508 patients were included in this report—225 in the group prior to the change in guidelines and 283 after. The incidence of birth via MSAF was similar between the two groups (11.2% in 2010–2015 vs. 11.5% in 2017–2022; *p* = 0.44). The baseline patient characteristics were similar between the two groups (Table 1).

No difference in the incidence of NICU admissions was noted, as shown in Table 2. Additionally, no statistically significant difference was observed in the incidence of endotracheal intubation, the use of mechanical ventilation, the use of non-invasive ventilation, the use of continuous positive airway pressure (CPAP), the use of oxygen, or the use of inhaled nitric oxide (iNO).

Furthermore, a significantly higher incidence of meconium aspiration syndrome (before 2016: 29.3% vs. after 2016: 19.7%; *p* = 0.03) and persistent pulmonary hypertension of the newborn (before 2016: 8.9% vs. after 2016: 2.5%; *p* = 0.003) was noted in the epoch prior to NRP guideline changes (Table 2). However, the 2017–2022 epoch was also noted to have a significantly higher number of neonates who were diagnosed with hypoxic–ischemic encephalopathy (HIE) and who received therapeutic hypothermia.

## 4. Discussion

In our institution, non-vigorous neonates born through MSAF who received tracheal suctioning in the delivery room had a significantly higher incidence of MAS (29.3% vs. 19.7%; *p* = 0.03) and PPHN (8.9% vs. 2.5%; *p* = 0.003). There was no difference in requiring extracorporeal membrane oxygenation (ECMO) or in the incidence of death before or after the change in NRP guidelines (0.4% vs. 1.1%; *p* = 0.45). The length of stay was similar between the groups (17.96 ± 1.88 prior to guideline change vs. 14.53 ± 1.44 after 2016; *p* = 0.14).

A significantly higher number of neonates in the non-suctioning era (2017–2022) were diagnosed with HIE (5.8% vs. 11.3%; *p* = 0.03) and received therapeutic hypothermia (3.6% vs. 10.2%; *p* = 0.006). It is uncertain whether this difference was observed due to an increased vigilance in diagnosing HIE or the introduction and modification of whole body cooling protocols as the decade progressed. Given the dynamic nature of neonatal management, it would be very difficult to gauge the true clinical implication of change in guidelines on birth asphyxia through a retrospective study.

Although the PPHN incidence significantly decreased in the second epoch, the use of inhaled nitric oxide after the change in guidelines remained similar (4.4% vs. 5.7%; *p* = 0.54). Since PPHN was well-defined and documented, when hypoxic respiratory failure was present in MAS patients with no echocardiographic features of PPHN, iNO was used based on a clinical suspicion for PPHN.

The neonatal resuscitation approach to MSAF has evolved over the last few decades. The seventh edition of the NRP guidelines (2015) eliminated routine endotracheal suctioning for non-vigorous infants born through meconium-stained amniotic fluid (MSAF), recommending that resuscitation follow the same principles as for births with clear fluid [6]. Initial steps should occur under a radiant warmer, with positive pressure ventilation initiated if the infant is apneic or the heart rate is <100 bpm. The revised recommendation was guided by the intent to reduce intubation-related risks and minimize delays in initiating PPV, given the lack of evidence supporting the benefit of routine tracheal suctioning. The eighth edition (2021) reaffirmed these recommendations, emphasizing that routine intrapartum suctioning offers no advantage [5].

The optimal management of non-vigorous infants born through MSAF remains debated. A meta-analysis by Dikou et al. evaluated six studies, with 1026 patients meeting inclusion criteria, and found no difference in the MAS incidence, length of hospitalization, or mortality between the two time periods [12]. A national database (Vermont Oxford Network) reported declining MAS rates following the NRP seventh edition guidelines [13,14]. A regional study, the California Perinatal Quality Care Collaborative (CPQCC), observed a significant decrease in the incidence of MAS in the no-suction era in NICUs across California [14]. We observed comparable results in our center.

After the guideline implementation, Aldhafeeri et al. reported no significant changes in MAS rates or complications related to MAS [15]. Myers et al. showed improved 1 min Apgar scores and reduced respiratory support needs with no notable change in NICU admission rates [16]. Oommenn et al. noted fewer NICU admissions since the change in guidelines [17].

Alternatively, one trial by Singh et al. evaluating 155 patients indicated a potential increase in MAS (41.3% to 57.1%) in the no-suction group (*p* = 0.052) [18]. Chiruvolu et al. documented increased respiratory NICU admissions, mechanical ventilation, oxygen therapy, and surfactant use, with a non-significant rise in MAS (5% to 11%) [19].

These differences between various centers may be due to multiple reasons, such as the different time frames of the two epochs with variable prenatal, labor, delivery, and NICU care and management; concomitant changes, like delayed cord clamping and umbilical cord milking; and differences in the resources, patient population, and skill sets among the providers, which may have played a role. Additionally, classifying a baby as non-vigorous can be very subjective, with significant variability between centers.

The large animal study of an ovine model from our lab showed that tracheal suctioning reduced the aspirated meconium density and improved oxygenation; however, PPV initiation was increased significantly by ~100 s. Though tracheal suctioning improved oxygenation and ventilation, it did NOT improve pulmonary/systemic hemodynamics or oxidative stress. Lung 3-nitrotyrosine levels were higher in the suction group (0.65 ± 0.03 ng/μg protein) compared to the no-suction group (0.47 ± 0.06 ng/μg protein), signifying more oxidative stress to the lung tissue [8].

Not spending time on tracheal suctioning and directly giving PPV could have been the reason we saw significantly less MAS and PPHN cases in our unit. Clearing the larger airways before the meconium reaches the lungs during initial breaths seems like a reasonable step in neonatal resuscitation. However, if deep gasping efforts before apnea have already caused in utero aspiration, this intervention may be ineffective. Additionally, intubation and tracheal suctioning can delay resuscitation, potentially worsening the hypoxic–ischemic injury in a compromised newborn.

Our current study has limitations. It is a single-center study with a relatively small sample size. The two-year gap between epochs may have introduced variability in acuity and management styles. MR.SOPA corrective steps (Mask adjustment, Reposition the airway, Suction the mouth and nose, Open the mouth, Pressure increase, and Alternative airway) were introduced in 2011, and patients born between 2010 and 2011 were accounted for in our study, but the effect of this intervention on our results is undetermined. We conducted a detailed chart review of all infants with MSAF, applying strict MAS criteria, which may have excluded mild cases. However, consistent guidelines were used across epochs to minimize misclassifications as transient tachypnea or other transitional disorders.

While our data shows a decreased incidence of MAS and PPHN in the non-suction group, other institutions (Karla et al. [1], Singh et al. [18], and Chiruvolu et al. [19]) reported higher numbers of MAS, PPHN, and the need for mechanical ventilation in the non-suctioning epoch. Lakshminrusimha et al. [8] observed improved oxygenation/ventilation parameters with suctioning. Most other cited studies showed either no difference or improvement in outcomes since the change in guidelines (non-suction group). Furthermore, there was an inexplicable rise in HIE rates after suctioning was discontinued in our institution, as reported. We speculate that this was due to the introduction of guidelines and protocols to start therapeutic hypothermia; however, there were many variables to consider and many practice changes over the years. Randomized controlled trials are strongly recommended to assess the true effects of the change in guidelines. Due to the relative rarity of this condition and the wide variety of results concerning this topic, we suggest a large multicenter trial comparing suctioning to not suctioning non-vigorous neonates born through MSAF to eliminate the variables in management protocols between two different epochs and yield unbiased answers for the best standard approach to this population of neonates.

## 5. Conclusions

In our institution, non-vigorous neonates born through meconium-stained amniotic fluid that did not receive endotracheal suctioning had a smaller incidence of MAS and PPHN. However, we also had a higher reported incidence of HIE and therapeutic hypothermia. Large-scale multicenter randomized controlled clinical trials may be needed to identify the best approach in managing non-vigorous neonates born through MSAF.

## Figures and Tables

**Table 1:** Patient characteristics.

Characteristics	2010–2015	2017–2022	*p*-Value
Infants born through MSAF	1339 (11.2%)	1473 (11.6%)	0.44
MSAF patients that were born non-vigorous (%)	225 (16.8%)	283 (19.2%)	0.71
Gestational age	39 3/7 ± 2 days	38 6/7 ± 6 days	0.13
Birth weight (kg)	3.28 ± 0.69	3.27 ± 0.65	0.77
Male gender	120 (53%)	133 (47%)	0.16
Multiple gestation	2 (0.9%)	6 (2.1%)	0.28
Apgar score 1 min [Median (IQR)]	4 (2–6)	3 (2–5)	0.32
Apgar score 5 min [Median (IQR)]	7 (6–8)	7 (6–8)	0.08
Apgar score 10 min [Median (IQR)]	8 (7–8)	8 (6–8)	0.21
Cord pH	7.16 ± 0.13	7.19 ± 0.12	0.07
C-section	132 (58.7%)	166 (58.7%)	0.99
Vaginal delivery	93 (41.3%)	117 (41.3%)	
General anesthesia for delivery	36 (16%)	40 (14%)	0.57
Spinal/epidural anesthesia for delivery	119 (53%)	153 (54%)	

**Table 2:** Outcomes for non-vigorous patients born through MSAF.

Outcomes	2010–2015 (n = 225)	2017–2022 (n = 283)	Odds Ratio (95% CI)	*p*-Value
Patients admitted to NICU	179 (80%)	241 (85%)	0.68 (0.43–1.08)	0.10
Patients diagnosed with MAS	66 (29.3%)	59 (19.7%)	1.58 (1.05–2.36)	0.03 *
Patients diagnosed with PPHN	20 (8.9%)	7 (2.5%)	3.85 (1.60–9.27)	0.003 *
Patients diagnosed with pneumothorax/pneumomediastinum	13 (5.8%)	20 (7.1%)	0.81 (0.39–1.66)	0.56
Patients diagnosed with Hypoxic–Ischemic Encephalopathy	13 (5.8%)	32 (11.3%)	0.48 (0.25–0.94)	0.03 *
Patients who received therapeutic hypothermia	8 (3.6%)	29 (10.2%)	0.32 (0.14–0.72)	0.006 *
Patients who received iNO	10 (4.4%)	16 (5.7%)	0.78 (0.35–1.75)	0.54
Patients who received ETT	73 (32.4%)	103 (36.4%)	0.83 (0.58–1.21)	0.35
Patients who received chest compressions	15 (6.7%)	14 (4.9%)	1.37 (0.65–2.91)	0.41
Patients who received epinephrine	4 (1.8%)	6 (2.1%)	0.84 (0.23–3.00)	0.78
Patients who received surfactant	17 (7.6%)	9 (3.2%)	2.49 (1.09–5.69)	0.03 *
Patients who received supplemental O_2_	69 (30.7%)	80 (28.3%)	1.12 (0.76–1.65)	0.56
Patients who received CPAP support	13 (5.8%)	7 (2.5%)	2.42 (0.95–6.17)	0.06
Patients who received non-invasive ventilation support	19 (8.4%)	20 (6.7%)	1.21 (0.63–2.33)	0.56
Patients who received conventional ventilator support	45 (20%)	44 (14.7%)	1.36 (0.86–2.15)	0.19
Patients who received high-frequency jet ventilator support	1 (0.4%)	2 (0.7%)	0.63 (0.06–6.96)	0.70
Patients who received high-frequency oscillator ventilator support	10 (4.4%)	8 (2.8%)	1.60 (0.62–4.12)	0.33
Patients who received mechanical ventilation	56 (24.9%)	54 (19.1%)	1.41 (0.92–2.15)	0.12
Mean ventilator days [per patient] ± SEM	2.27 ± 0.3	2.26 ± 0.32		0.98
Median ventilator days [per patient] (IQR)	2 (1–2)	2 (1–3)		
Patients who received non-invasive mechanical ventilation (%)	101 (44.9%)	107 (37.8%)	1.34 (0.94–1.91)	0.11
Mean days on non-invasive respiratory support [per patient] ± SEM	5.31 ± 2.36	4.62 ± 0.65		0.65
Median days on non-invasive respiratory support [per patient] (IQR)	2 (1–4)	2 (1–5)		
Number of patients requiring ECMO	0 (0%)	2 (0.4%)	0.25 (0.01–5.26)	0.37
Number of patient deaths (%)	1 (0.4%)	3 (1.1%)	0.42 (0.04–4.03)	0.45
Length of stay	17.96 ± 1.88	14.53 ± 1.44		0.14

CPAP: Continuous positive airway pressure. ECMO: Extracorporeal membrane oxygenation. ETT: Endotracheal tube. iNO: Inhaled nitric oxide. IQR: Interquartile range. MAS: Meconium aspiration syndrome. MSAF: Meconium-stained amniotic fluid. NICU: Neonatal intensive care unit. PPHN: Persistent pulmonary hypertension of the newborn. SEM: Standard error of mean. * *p*-value < 0.05.

## Data Availability

The raw data supporting the conclusions of this article will be made available by the authors on request due to due to privacy and ethical restrictions.

## Abbreviations

The following abbreviations are used in this manuscript:

CPAP	Continuous positive airway pressure
ECMO	Extracorporeal membrane oxygenation
ETT	Endotracheal tube
HIE	Hypoxic–ischemic encephalopathy
iNO	Inhaled nitric oxide
IQR	Interquartile range
MAS	Meconium aspiration syndrome
MSAF	Meconium-stained amniotic fluid
NICU	Neonatal intensive care unit
NRP	Neonatal resuscitation program
PPHN	Persistent pulmonary hypertension of the newborn
PPV	Positive pressure ventilation
SEM	Standard error of mean
